# Validation study on a prediction formula to estimate the weight of children & adolescents with special needs aged 2–18 years old

**DOI:** 10.1186/s41043-023-00464-5

**Published:** 2023-11-20

**Authors:** Nurul Huda Ibrahim, Norasimah Kassim, Salimah Othman, Azahadi Omar, Norsuhaila Shaari, Anis Aslah Awiskarni, Norafidza Ashiquin Abdul Patah, Nabila Mohamed Nezuri, Maizatul Naqiah Zulfifli, Mohd Nadzrul Anuar Awang, Muhamad Farid Sani, Noorfadzlina Abdul Rashad, Siti Farhana Mesbah

**Affiliations:** 1 Institute for Public Health, Ministry of Health Malaysia, 40170 Setia Alam, Selangor Malaysia; 2 Family Health Development Division, Ministry of Health Malaysia, 62590 Putrajaya, Malaysia; 3 National Institute of Health, Ministry of Health Malaysia, Setia Alam, Malaysia; 4 Hospital Kuala Lumpur, Kuala Lumpur, Malaysia; 5 Klinik Kesihatan Bandar Botanik, Ministry of Health, 42000 Klang, Selangor Malaysia; 6 Klinik Kesihatan Seremban, Seremban, Malaysia; 7 Klinik Kesihatan Nilai, Nilai, Negeri Sembilan Malaysia; 8 Klinik Kesihatan Seksyen 19, Shah Alam, Selangor Malaysia; 9 Klinik Kesihatan Putrajaya, Persint 18, Putrajaya, Malaysia; 10 Klinik Kesihatan Cheras, Kuala Lumpur, Malaysia

**Keywords:** Children with special needs, Body weight estimation, Body weight formula, MUAC

## Abstract

**Background:**

This study aims to validate two predictive formulas of weight estimating strategies in children with special needs, namely the Cattermole formula and the Mercy formula.

**Methodology:**

A cross-sectional study with a universal sampling of children and adolescents with special needs aged 2–18 years old, diagnosed with cerebral palsy, down syndrome, autism and attention-deficit/hyperactivity disorder was conducted at Community-Based Rehabilitation in Central Zone Malaysia. Socio-demographic data were obtained from files, and medical reports and anthropometric measurements (body weight, height, humeral length, and mid-upper arm circumference) were collected using standard procedures. Data were analysed using IBM SPSS version 26. The accuracy of the formula was determined by intraclass correlation, prediction at 20% of actual body weight, residual error (RE) and root mean square error (RMSE).

**Result:**

A total of 502 children with a median age of 7 (6) years were enrolled in this study. The results showed that the Mercy formula demonstrated a smaller degree of bias than the Cattermole formula (PE = 1.97 ± 15.99% and 21.13 ± 27.76%, respectively). The Mercy formula showed the highest intraclass correlation coefficient (0.936 vs. 0.858) and predicted weight within 20% of the actual value in the largest proportion of participants (84% vs. 48%). The Mercy formula also demonstrated lower RE (0.3 vs. 3.6) and RMSE (3.84 vs. 6.56) compared to the Cattermole formula. Mercy offered the best option for weight estimation in children with special needs in our study population.

## Introduction

Body weight is a crucial anthropometric measure that health care professionals frequently use to analyse children's growth charts and developmental patterns. In clinical and community settings, body weight is an important indicator for dietary and medical intervention. Several techniques have been developed to assess body weight in children who are typically developed (TD). Children with both physical and intellectual disabilities lacked the validity of the body weight predictive equation. These distinct individuals could have varying morphologies, body types, and distinctive traits that are compromised [1].

Nutritional status is an important indicator of the overall health status and well-being of disabled children [2]. Low nutritional status was a prime contributor to poor-growth children [3]. Malnutrition is common in children with special needs due to feeding difficulties [4–6], frequent illness [5], poor nutrient absorption [7] and poor care [8]. Children with cerebral palsy (CP) and Down syndrome (DS) are at risk of nutritional deficiency due to poor oral motor feeding and swallowing problems [9].

In Malaysia, the prevalence of underweight ranged from 22.2 to 78.2% among children with CP. Children with DS are at risk for overweight and obesity, with a prevalence of 33.5–43.5% [2]. With a prevalence of 7.8% underweight and 24.8% overweight or obese, children with autism spectrum disorder (ASD) are at risk for double-burden malnutrition [2].

Using a calibrated weighing scale is the gold standard for determining a child's weight [10]. Various methods have been established for measuring body weight in children who are typically developed (TD). These distinct individuals could have varying morphologies, physiques, or other distinctive traits [1]. Due to their oppositional behaviour and physical impairments, children with special needs may make it challenging to gauge their body weight. Thus, alternative weight equations/formulas are required to estimate weight in this unique group. According to other research [11, 12], body length/height, humeral length (HL) and mid-upper arm circumference (MUAC) are the usual alternative measurements for weight in children. A weight estimation formula includes at least one of the alternate metrics given. Several methods of paediatric weight estimation were developed based on age, body length and MUAC. In particular, the Broselow emergency tape was frequently utilised in Malaysia to estimate the body weight of paediatric patients in the emergency room based on their length and height [13]. However, the Broselow method could not be applied to children with severe joint contractures or neurologic defects [13, 14]. In addition, most age-based weight estimation methods, such as Advanced Pediatric Life Support (APLS), are restricted to healthy children up to 15 years only [11]. Hence, this study only involves length and habitus-based weight estimation methods, namely the Cattermole (MUAC) and Mercy (Humerus Length & MUAC) methods. These methods could be applied in children with special needs regardless of their age and height limitations.

It is still unclear how accurate weight assessment is in children with special needs (CWSNs). Only one study estimated weight in children with Down syndrome [15]. To date, no published research has been conducted to evaluate the performance of weight estimating methods in CWSNs in Malaysia.

This research was therefore critical in attempting to determine an accurate method of weight estimation among CWSNs. Hence, this study was conducted to validate two predictive formulas of weight estimation strategies in children with special needs, namely the Cattermole (MUAC) formula and the Mercy (HL and MUAC) formula. Furthermore, this study could help health care practitioners estimate weight whenever direct weight measurement is impossible or impractical.

## Methodology

### Study design and population

A cross-sectional study design with universal sampling was conducted at selected Community-Based Rehabilitation Centres (CBRs) in Central Zone Malaysia (Selangor, Federal Territory of Putrajaya and Kuala Lumpur and Seremban). Children diagnosed with Down Syndrome (DS), Cerebral Palsy (CP) and autism spectrum disorder (ASD)/Attention Deficit Hyperactive Disorder (ADHD) aged 2 to 18 years who attended Community-Based Rehabilitation Centres (CBR) were included in this study. Meanwhile, children diagnosed with other disabilities and suffering from oedema, ascites, pleural effusion, large tumour condition and hydrocephalus were excluded from the study. Study protocol approval was obtained from the National Medical Research Registry Malaysia (NMRR) (ID: NMRR-17-2743-35970). The ethics of the protocol was approved by the Medical Research and Ethics Committee (MREC) Malaysia with confidentiality maintained for all subjects. Written consent was obtained from the parent/caregiver prior to data collection. Out of 3,436 children who enrolled in CBR, 502 children met the inclusion criteria and consented to participate in this study.

### Data collection

Socio-demographic data were obtained from individual files, and medical reports and anthropometric measurements were performed for body weight, height, humeral length (HL) and mid-upper arm circumference (MUAC). The actual body weight of the children was measured by a SECA 674 platform weighing scale in any position, either standing upright, sitting, or lying down, and readings were recorded to the nearest 0.01 kg. Body height was measured with a standard procedure using a stadiometer SECA 213 to the nearest 0.1 cm [16]. Humeral length and MUAC were measured using the retractable measuring tape SECA 201 to the nearest 0.1 cm. HL was measured from the top of the shoulder (acromion) to the point of the elbow (olecranon process) [17], while MUAC was measured at the midpoint of the distance between the shoulder bone (acromion) and the elbow (electron process). Data on HL and MUAC were applied to the Mercy equation, while MUAC alone was applied to the Cattermole equation [(MUAC in cm − 10) × 3] [18].

### Statistical analysis

All data obtained were analysed using IBM SPSS (Statistical Package for Social Science) version 26. The normality of the continuous data was determined by using Shapiro‒Wilk’s and Kolmogorov‒Smirnov tests. Demographic data are presented as frequencies and percentages for categorical variables. Meanwhile, the mean ± standard deviation (SD) or median ± interquartile range (IQR) was described in the continuous data. Spearman correlation was used to determine the relationship between variables. The mean error (ME), mean percentage error (MPE) and root mean square error (RMSE) were determined. Residual error (RE) was calculated by taking the difference in estimated weight and actual weight. Next, percentage error (PE) was calculated by dividing the actual weight into the mean error (ME) and multiplying by 100. The root mean square error (RMSE) was calculated by taking the square root of the average squared error. The percentages of estimated weights and actual weight are targeted at the level of 20% from actual weight by analysis of Variance (ANOVA) and Bland‒Altman plots. In addition, the intraclass correlation (ICC) was calculated using a 2-way random-effects model, and absolute agreement with a significant level was determined at *p* < 0.05.

## Results

A total of 502 special needs children aged 2 to 18 years participated in this study. The median age of the children was 7 (6) years; two-thirds of them (66.7%) were boys, and the majority were of Malay ethnicity (91.2%). Approximately, 31.5% of the studied population was diagnosed with cerebral palsy, and half of them were in GMFCS I to GMFCS III. Detail socio-demographic as presented in Table 1.

**Table 1:** Socio-demographic data

Characteristic	*n* (%)	Median (IQR)
Age (year)		7.0 (6)
Gender		
Male, *n* (%)	335 (66.7)	
Female, *n* (%)	167 (33.3)	
Ethnicity		
Malay	458 (91.2)	
Chinese	21 (4.2)	
Indian	14 (2.8)	
Others	9 (1.8)	
Diagnosis		
Down syndrome	142 (28.3)	
ADHD/Autism	202 (40.2)	
Cerebral palsy	158 (31.5)	
GMFCS I	26 (16.6)	
GMFCS II	33 (21.0)	
GMFCS III	26 (16.6)	
GMFCS IV	9 (5.7)	
GMFCS V	63 (40.1)	

Anthropometric measurements are stated in Table 2. Body height, mid-upper arm circumference and humeral length showed a strong correlation with body weight, as presented in Table 2.

**Table 2:** Anthropometric measurement and correlation with body weight

	Median	Min	Max	*r*	*p*
Body weight (kg)	18.27	7.65	94.65	–	–
Height (cm)	111.1	72.8	172.5	0.916	0.001
Mid-upper arm circumference (cm)	17.4	12.2	37.0	0.858	0.001
Humeral length (cm)	22.2	12.6	37.9	0.850	0.001

**Fig. 1:**
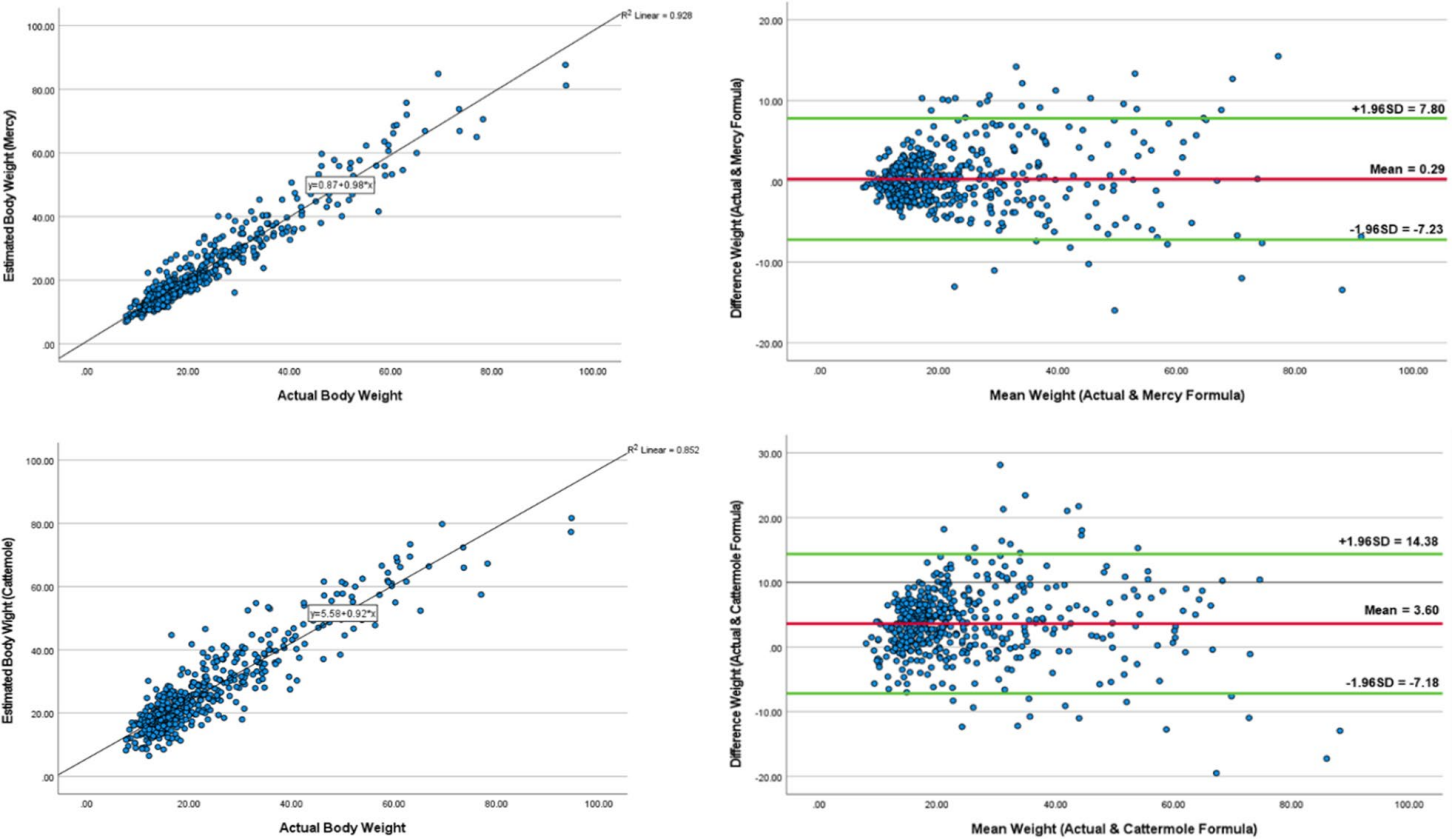
Regression between the predictive equation and actual weight and degree of agreement

The results show that the Mercy formula demonstrated a smaller degree of bias than the Cattermole formula (PE = 1.97 ± 15.99% and 21.13 ± 27.76%, respectively). The Mercy formula showed a higher intraclass correlation coefficient (0.936 vs. 0.858) and predicted weight within 20% of the actual value in the largest proportion of participants (84% vs. 48%). The Mercy formula also demonstrated lower RE (0.3 vs. 3.6) and RMSE (3.84 vs. 6.56) compared to the Cattermole formula. Figure 1 shows the regression between predictive weight and actual weight and the Bland‒Altman plot for both predictive equations.

The predictive performance of the Cattermole and Mercy weight estimation methods are shown in Table 3.

**Table 3:** Predictive performance of Cattermole and Mercy methods

	Cattermole	Mercy
Eligible sample size	500	498
Agreement within 20% of actual	48.2%	84.5%
Residual error (kg)	3.599 ± 5.497	0.287 ± 3.833
Percentage error (%)	21.134 ± 27.763	1.966 ± 15.985
Root-mean-square error (RMSE)	6.56	3.84

## Discussion and conclusion

This study offered important insight into weight estimation for children with special needs aged 2–18 years whenever the standard weight measurement was impossible. Due to the physical constraints and cognitive disability among children with special needs, alternative weight measurements are required to predict their actual weight. In this study, the Cattermole and Mercy weight estimation methods were comparable, and surprisingly, both methods were predictors of the actual weight. The Mercy weight estimate method (partial weight of HL + partial weight of MUAC) was more accurate in predicting the real weight than the Cattermole weight estimation method. Findings from this study were in agreement with a previous study conducted by Talib et al. [15] in the United States of America, which demonstrated Mercy as the best option to estimate weight in Down syndrome children aged 18 years and below compared with other methods, including the Cattermole, Broselow tape and APLS methods.

In addition, the agreement within 20% of the actual weight between the current study and the previous study [15] was comparable. The Mercy method in this study demonstrated the closest proportion of children’s estimated weight within 20% of actual weight with the Mercy method in a previous study (85% vs. 88%). Similarly, the Cattermole method in this study proposed a near proportion of children’s estimated weight within 20% of actual weight with the Cattermole method in the previous study (48% vs. 40%). Nevertheless, the Mercy method in the current study was found to overestimate the actual weight, while the Mercy method in the previous study [15] was reported to underestimate the actual weight (RE 0.287 kg vs. − 1.4 kg).

The Mercy method performed better than the Cattermole method in this study because the two-dimensional systems were always far superior in accuracy to the one-dimensional system [19]. The Mercy method incorporates two-dimensional systems (HL and MUAC), which results in more accurate weight estimation than the Cattermole method, which only relies on MUAC [15, 11, 19]. To date, no study has reported that one-dimensional systems are more accurate than two-dimensional systems [19].

Furthermore, the finding of low accuracy of the Cattermole method in this study was in line with the studies conducted among Korean children by Choi et al. [21] and Suh et al. [20], which revealed that the Cattermole method was the worst accurate method and was only highly precise in children aged 6 to 14 years. Moreover, the accuracy of the Cattermole weight estimation method to predict weight in children with special needs is unclear. The existing studies related to Cattermole estimation weight were conducted among normal children [18, 21, 20] and were reliable in school-age children [18].

Instead of special needs children, the Mercy weight estimation method was also accurate in predicting weight in normal children [22–25]. Additionally, the Mercy approach proved more accurate than other methods in a wider range of ages [11]. Hence, it has been observed that the Mercy weight estimation method was more applicable in both normal and special needs children with a wide age range.

Several limitations to this study need to be acknowledged. This study did not engage with normal children or other special needs children diagnosed beyond Down syndrome, autism/ADHD and cerebral palsy. In addition, the current study merely focused on children with special needs aged 2–18 years, and findings from this study could not be extrapolated to other age populations, such as adults and elderly individuals. Moreover, the accuracy of weight estimation methods decreases with increasing age [23]. Furthermore, this study only involved two weight estimation methods, namely the Cattermole and Mercy methods, that could predict the weight of the children in this study. The accuracy of other weight estimation methods could not be determined in the current study. In addition, there is no reference standard or benchmark for assessing the accuracy of the weight estimation methods. Therefore, further local research is required to explore the accuracy of other weight estimation methods.

In conclusion, the humeral length and mid-upper arm circumference were the most robust factors for predicting actual weight in children with special needs. The most apparent finding that emerged from this study was that the Mercy weight estimation method performed well in Malaysian special needs children, similar to that shown in the Western population. Hence, the Mercy weight estimation method is recommended to predict the actual weight in Malaysian special needs children aged 2 to 18 years.

## Data Availability

The datasets used and/or analysed during the current study are available from the corresponding author on reasonable requests.

## Abbreviations

MUAC: Mid-upper arm circumference
HL: Humeral length
GMFCS: Gross motor function classification system
CP: Cerebral palsy
DS: Down syndrome
CWSN: Children with special needs
